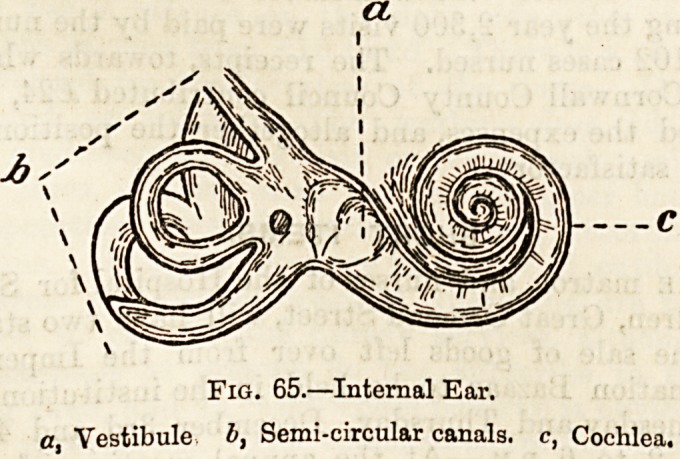# The Hospital. Nursing Section

**Published:** 1902-11-15

**Authors:** 


### The Hospital.

### Nursing Section.

Contributions for this Section of "THE HOSPITAL" should be addressed to the EDITOR, "THE HOSPITAL" NURSING SECTION, 28 & 29 Southampton Street, Strand, London, W.C.

No. 842.—VOL. XXXIII. *SATURDAY, NOVEMBER* 15, 1902.

### Notes on News from the Nursing World.

### THE ROYAL RED CROSS AND ITS RECIPIENTS.

IN another column "An Onlooker" gives expression to the discontent which appears to prevail amongst some nurses respecting the manner in which the decoration of the Royal Red Cross is bestowed. We publish the letter because it serves to show the misapprehensions that prevail upon the subject. The order is not, as our correspondent imagines, by any means intended for nurses only. It was instituted on St. George's Day 1883, to be conferred "for zeal and devotion in providing for and nursing sick and wounded sailors, soldiers, and others with the army in the field, on board ship, or in hospitals." In the list of 144 who possessed the order at the end of last year, more than half are either matrons, superintendents, or sisters, and recent additions have certainly not affected the balance. We imagine that every nurse rejoices that the Queen and other members of the Royal Family attest their value of the decoration by receiving it; and it will scarcely be contended that the Dowager Lady Dudley, Lady Sarah Wilson, Lady Roberts, and others have not earned it "by providing for" the sick and wounded. Of course, it may be true that some claims have been overlooked, and that there have been selections which were not anticipated; but we do not think that there is any solid ground for the charge of favouritism. No exception will, assuredly, be taken to the distinction which it was announced on Monday, in the King's list of Birthday Honours, has been conferred upon Mrs. Crowlie. This brave and devoted lady has rather tardily been awarded the Royal Red Cross in recognition of her services to the sick and wounded seamen and marines in the Tientsin Hospital during the operations against the "Boxer" rebels in June and July 1900.

### A GIFT BY MISS FLORENCE NIGHTINGALE.

THE interest which Miss Florence Nightingale in her retirement continues to show in all movements for the benefit of the community, has again been manifested by her handsome gift to a Buckinghamshire institution. Miss Nightingale has sent a cheque for £50 towards providing books for Steeple Claydon Library. The committee have had the cheque framed and hung on the wall beneath the portrait of the donor.

### TESTIMONIAL TO THE LATE MATRON OF ST. THOMAS'S HOSPITAL.

MISS GORDON, on her retirement from St. Thomas's Hospital, has been presented with a testimonial from her past and present nurses. The subscription was limited, but the long list of subscribers, including names from many parts of the world, shows the esteem and affection in which she is held by those who have worked under her. The testimonial, at Miss Gordon's request, is to be used towards the furnishing of her house.

### OUR CHRISTMAS DISTRIBUTION.

THE receipt of the usual appeals from matrons of hospitals and infirmaries in various parts of the metropolis where the population is dense and poverty-stricken, is a sufficient reason for drawing attention again to our Christmas distribution. On this occasion we are anxious, in view of the exhibition, to chronicle a record, and we appeal with confidence to our readers to enable us to do so. In addition to the contributions already acknowledged we have received parcels from :—Nurse Allen, care of Miss Sands, Dunan, Aldeburgh, Suffolk; M. R., Ryde; Nurse A. Medforth, 7 Colleton Crescent, Exeter; A "Co." Nurse, London; and Miss Helen Grafton, Wessington Court, Woolhope, Hereford. They should in all cases be addressed to the Editor, 28 & 29 Southampton Street, Strand, London, W.C., and should be marked "Clothing Distribution."

### WHOLESALE DISMISSAL OF NURSES.

WE are informed that fourteen members of the nursing staff attached to the General Hospital, Cheltenham, or the private nursing staff in connection with it, have received notice of dismissal without a hearing from the committee. The offence alleged against them is that these nurses drew up and forwarded to the authorities a petition asking that their salaries might be made equivalent to the salary of a probationer who, with less experience, has recently been appointed a ward sister. It is stated that although there is nothing against the character, or the efficiency, of the nurses in question, they are not to receive a testimonial. Assuming the accuracy of this information, the case is one that clearly calls for explanation.

### NURSES FROM SOUTH AFRICA.

THE following nurses have arrived from South Africa since the publication of our last issue :—On the *Dunvegan Castle* Sisters C. Condell, E. C. Lloyd, B. F. Whyte, E. A. Howard, A.N.S.R.; on the *Orient*, Sisters R. Beaufoy, L. F. Bristow, A.N.S.R., and F. Matchett, New South Wales Nursing Service.

### LEAGUE OF ST. JOHN'S HOUSE NURSES.

A GENERAL meeting of the League of St. John's House Nurses was held at St. John's House, Norfolk Street, Strand, on Thursday last week. A large number of members were present. Minutes of the last general meeting in May were read and confirmed. Although yet in its early youth, the League has done some good work. The self-instruction scheme suggested by one of the members has been well responded to. The subjects chosen are botany, literature, and French. French evidently has the preference, probably because so many nurses have felt the need of a knowledge of it in private nursing, not only abroad, but also often at home. Three very good collections of flowers were sent in for inspection by members of the botany group. The magazine published half-yearly under the title of "St. John's House News" is much appreciated, and has already travelled to all four quarters of the globe. A small sum has been set aside as a nucleus for a delegate fund, and a small annual subscription has also been voted to the Society for the State Registration of Trained Nurses. The League now numbers 95 members. The meeting was followed by a social gathering of old friends, many of whom came from a distance, and among other guests the president of the Chelsea Infirmary League and the late secretary of the League of St. Bartholomew's gave much pleasure by their attendance.

### THE NURSES' HOME AT LAMBETH INFIRMARY.

Last week the foundation stone of the set of buildings adjoining the Lambeth Workhouse Infirmary, and intended for use as a nurses' home and offices was laid by Mr. George Howlett, Chairman of the Board of Guardians, in the presence of a large company. The Home, which is to have accommodation for nearly 100 nurses, will face Brook Street, Kennington Road, and the ceremony took place at the north-east angle of the new board room. At present two houses have to be rented at £96 a year for the accommodation of the nurses, and this amount will, of course, be saved when the home is finished. The estimated cost of the new buildings is about £4,000.

### A GRADUATED FEE FOR NURSING.

The Fishponds District Nursing Association has just completed its second year of existence. The report shows that the nurse paid 2,273 visits during the year ended August 31, and after paying its way so far has a balance on the right side; the subscriptions varying from £10 to 2d. There is a small fee charged for the services of the nurse. It is graduated as follows:—The working-class, 1d. a visit; tradespeople, 1s. a visit, or 15s. a week; midwifery cases, not less than 6s.; massage, per hour, 5s.; operation cases, in patient's home, according to means. Patients who are in receipt of outdoor relief from the Bristol Board of Guardians, are not expected to pay for the nurse's services, although the Guardians at present do not subscribe to the nursing fund, and in cases where poverty is obvious no fee is expected. The committee are fortunate in commanding the services of a Nightingale trained nurse, who was staff nurse at St. Thomas's Hospital, London, for several years.

### CROYDON INFIRMARY.

At the meeting of the Croydon Board of Guardians on Tuesday the Infirmary Committee reported that they had received and adopted the following special report of the visitors of October 10th, namely:—"We interviewed 17 of the nurses, whose signatures are appended to the complaint referred to us by the Visiting Committee as to the training of nurses at the Infirmary. No specific charges of deficient or inefficient training were made, but only general charges not amounting to deficiency or inefficiency in training. Exception was taken to the form of certificate, but no instance of hardship or inconvenience was brought forward. We explained the reason of the present form of certificate, and expressed an opinion that the apprehensions are not based upon facts and are groundless." The report was adopted.

### RESIGNATION OF A MATRON AND STAFF.

We learn that the matron and staff of the Folkestone Sanatorium have resigned. There must, of course, have been a reason for this very extreme step, and further details will be awaited with interest. The matron was trained at the London Hospital, and has the reputation of being, not only highly qualified for her position, but exceedingly capable.

### AN INCREASED DEFICIT AT BRIGHTON.

At the annual meeting of the Brighton, Hove, and District Nursing Association, over which the vicar presided, a very unsatisfactory report, from a financial point of view, was read. In consequence of the lack of funds, the committee were reluctantly compelled last March to give up five staff nurses. Yet, even after reducing the expenditure so considerably, the balance on the wrong side at the end of the financial year was £705 12s. 10d., as against £462 17s. 7d. the preceding year. This, it was stated in the report, is in spite of the fact that the committee have "again and again appealed for more generous support." The record of the nurses shows how essential their work is. With a staff of seven, including the superintendent, upwards of 1,000 cases were nursed in 12 months, and nearly 37,000 visits paid. The chairman told the meeting that 18 nurses are required to supply the needs of the district, and an income of about £1,800. This, he said, would be about 2½d. per head per annum; and allowing for the number of people who cannot, perhaps, afford to give anything, it will be a disgrace to Brighton if the income of the Association, which was only £1,300 last year, cannot, by means of persistence in asking and ordinary liberality in giving, be augmented to the extent of £500. As Canon Hoskyns put it, 2½d. is not a great tax for a charity purely local, appealing to all classes and denominations, and managed on lines of strict economy.

### THE MIDWIVES' BOARD.

The constitution of the Central Midwives' Board is now nearly complete. The following representatives have already been nominated: Dr. F. H. Champneys, by the Royal College of Physicians; Mr. J. Ward Cousins, by the Royal College of Surgeons; Dr. Parker Young, by the Society of Apothecaries; Dr. C. J. Cullingworth, by the Incorporated Midwives' Institute; Mr. J. Heywood Johnstone, by the Association of County Councils; Miss Rosalind Paget, by the Queen Victoria's Jubilee Institute; and Miss Oldham, by the Royal British Nurses' Association. The Lord President of the Council has still to appoint two members, one of whom must be a woman.

### NO QUALIFIED NURSE FOR KINGSCLERE.

In spite of the recommendation of the Medical Officer and the Inspector of the Local Government Board, the Kingsclere Board of Guardians have refused to move with the times and appoint a trained nurse at the Workhouse Infirmary. It is fair to say that two members of the board declared themselves in favour of the recommedation, but the majority took the view that there was no need to entail the expenditure of £50 a year which the engagement of a trained nurse would involve. "Provision," it was stated, "is made for securing a nurse in sudden and urgent cases." But this makeshift—which may any day fail—is an acknowledgment of the folly of the decision arrived at.

### DISTRICT NURSING AT REDRUTH.

The committee of the Redruth District Nursing Association report that from November 1st, 1901, to the same date this year, 3,564 visits were paid to about 100 patients, sufferers, in some instances, from painful and serious diseases. Surgical operations have also been attended when necessary. A contribution of two guineas from the Redruth Football Club indicates to appreciation of the work, as well as £3 8s. 2d. contributions for special services, and grateful letters from persons nursed and their friends. The total receipts, including balance in hand at the beginning of the year, were £102, and total expenditure £74. Mrs. A. Lanyon, who eight years ago founded the association, and has since acted for eight years as treasurer and secretary, now resigns the post of secretary, but retains that of treasurer, Mrs. Edwards and Miss Lanyon acting as joint secretaries. At the committee meeting a hearty and unanimous vote of thanks was awarded Mrs. Lanyon for her valuable help in the past.

### A DANGER SIGNAL AT ALFRETON.

It is always unsatisfactory when an organisation which has had a vigorous life begins to show signs of drooping. At the time the Alfreton Trained Nurse Fund was started, twelve years ago, the movement had not taken root in that part of Derbyshire, but since then nursing associations have been formed in several adjacent districts. It would be a sad pity if Alfreton fell back into the rear, and we welcome the assurance of one of the speakers at the annual meeting of the subscribers to the Trained Nurse Fund "that if the people of the town once realised that there was any danger of the movement coming to an end they would be up in arms and ready to support it immediately." As a diminishing balance should always be regarded in the light of a danger signal, it is not too soon for the people of Alfreton to wake up and put the Nurse Fund, which they admittedly value so much, on a sound footing.

### A PALMIST'S NOTION OF A GOOD HOSPITAL NURSE.

At the West London Police Court, on Monday, a foreign palmist was charged with telling fortunes. One of the witnesses, a young lady residing in Shepherd's Bush, said that she went to consult him, and that at his request she placed her hand flat on the table. He observed that "she had a strong will, was distrustful, and had a bad temper;" but she would make "a good hospital nurse."

### ASYLUM ATTENDANTS AND REFERENCES.

In the course of evidence given at Stratford Police Court on Saturday in support of a charge of assault against an attendant now at Horton Asylum, Epsom, but formerly at West Ham Borough Lunatic Asylum, it was stated by the deputy Town Clerk of West Ham that no inquiry was made at the institution at Chadwell Heath respecting the attendant by the authorities at Epsom. The charge against the accused was dismissed by the bench, but the chairman expressed his surprise that persons should be taken on at any lunatic asylum without asking for a reference from their last place. Undoubtedly, it is hopeless to attempt to improve the status of asylum attendants, or to induce a better class to take up the work so long as such looseness is practised by those in power.

### SUTHERLAND BENEFIT NURSING ASSOCIATION.

In the seventh annual report of the Sutherland Benefit Nursing Association there is the following passage :—"It is always the aim of the Association to secure for training women, who have a real liking for the work. Without this a nurse never attains success, in whatever grade or sphere of work she may be employed." This is a wise principle, and it is satisfactory to know that, as a rule, the Sutherland nurses have been proved to possess the requisite qualifications. During the year ending July 31, 1902, 259 cases were nursed by the members of the staff, and it is stated that the value of their services is recognised by the medical men, and valued by the patients and their relatives. A feature of the accounts, which are admirably set forth, is an abstract showing the revenue and expenditure of the twelve parishes.

### THE DISTRICT NURSE AT HELSTON.

The pleasant little town of Helston, in Cornwall, has had its district nurse for five years. In the report which has just been adopted at the annual meeting, an appropriate reference is made to the loss sustained by the death of the president, Mrs. Tyacke, and of another valued member of the committee. During the year 2,300 visits were paid by the nurse, and 102 cases nursed. The receipts, towards which the Cornwall County Council contributed £24, exceeded the expenses, and altogether the position is quite satisfactory.

### SHORT ITEMS.

The matron and nurses of the Hospital for Sick Children, Great Ormond Street, will have two stalls at the sale of goods left over from the Imperial Coronation Bazaar to be held in the institution on Wednesday and Thursday, December 3rd and 4th, from 2 to 6 P.M.—At the annual meeting of the Leven Nursing Association, N.B., it was agreed to include in the report an appreciation of the services of the late Miss M'Dougal. It was mentioned as an interesting fact that shortly before her death Miss M'Dougal had received a gratuity of £5 from the Jubilee Institute, a sum given to a certain number of Queen's nurses in order of seniority, the council recognising in this way the work of those who serve the Institute long and well.

### Lectures to Nurses on Anatomy.

By W. Johnson Smith, F.R.C.S., Principal Medical Officer, Seamen's Hospital, Greenwich.

### LECTURE XXX.—ORGANS OF SPECIAL SENSE (*Continued*).

*Hearing*.—The apparatus of this special sense, the complexity of which is indicated by the term of *labyrinth* given to one of its divisions, consists of (1) the *external*, (2) the *middle*, and (3) the *internal ear*. The aerial waves of sound set in motion by the vibration of a sound-producing body, just as waves are formed around a stone falling on

Fig. 64.—Section of the Ear.

smooth water, are collected and concentrated in the external ear, are then transmitted through membrane, bone, and fluid, and finally impinge on a membrane immersed in fluid, and are there converted at the minute and spread-out ends of the filaments of the eighth nerve into auditory impulses.

The external and the middle ear are but accessory parts; the structures of the internal ear enclosed in a spiral

Fig. 65.—Internal Ear.
*a*, Vestibule *b*, Semi-circular canals. *c*, Cochlea.

chamber within the petrosal portion of the temporal bone constitute the essential part. Should the two former be damaged by disease sound may still be transmitted to the intact internal ear by the bony walls of the skull. If the external ear be closely stopped by the tip of a finger we can still perceive the vibrations of a tuning-fork applied to the top of the head.

*The External Ear* (fig. 64).—The crumpled sheet of cartilage forming what is commonly called the ear, but which is described by anatomists as the *pinna* or *auricle*, presents several constant features, as, for instance, the marginal thickening, the *helix* (*a*), the swollen mass at the lower extremity, the *lobule* (*c*) and the deep central cavity, the *concha* (*b*) overhung in front by the hairy *tragus* and behind by a smaller projection, the *anti-tragus*. At the bottom of the concha is the entrance or *meatus* to the *auditory canal* (*d*), which runs inwards and a little forwards to a thin membrane, the membrana tympani (*e*), which at a distance of a little more than an inch from the surface separates the external ear passages from the middle ear or *tympanum* (*g*).

*The Middle Ear or Tympanum* is a small air-cavity in the petrosal bone, which is closed on the outside, as we have seen, by the membrana tympani, and on the inner and opposite surface by a much smaller membrane stretched over an inlet to the internal ear called the *fenestra ovalis* or oval window. Extending from the one membrane to the other is a chain of three minute bones, *the ossicles of the ear*. The external one, that in contact with the membrana tympani, is called the *malleus*, from its supposed resemblance to a hammer, the middle one, the *incus*, from its supposed resemblance to an anvil, and the third one, which is in contact with the membrane shutting in the internal ear, from its decidedly close resemblance in form to a stirrup, is called the *stapes*. On the front wall of the tympanum is the opening of the *Eustachian tube* (*f*), which opens at its lower end behind the nostrils and serves to supply the tympanum with air. At the upper part of the posterior wall is a communication with the large mastoid cell known as the *mastoid antrum*. A thin layer of bone separates the tympanum from the base of the brain, and the floor of the cavity is in close vicinity to two large vessels, the jugular vein and the internal carotid artery.

Passing beyond the tympanum we reach the internal ear, and find there a very intricate arrangement of chambers and canals hollowed out of the rocky portion of the temporal bone. You will, I fear, find much difficulty in gaining a correct idea of this—the essential part of the organ of hearing—unless in following any description or delineations of it you are assisted by a large and good model.

The posterior link of the chain of ossicles—the stapes or stirrup bone—is, as we have just seen, attached to a thin membrane stretched across and completely closing an opening on the inner wall of the tympanum—the fenestra ovalis. On the other side of this membrane there is a small oval chamber measuring about a quarter of an inch from floor to roof, and a fifth of an inch from side to side (*i*). On the back wall of this important chamber, which, we should remember, is called the *vestibule*, there are five minute round openings which lead into the three *semi-circular canals* (*k*), narrow curved tunnels, each describing a little more than a half circle, the relative positions of which are indicated by the distinguishing titles: *superior vertical canal, posterior vertical canal*, and *external horizontal canal*. One end of each of these three canals is slightly dilated into a bulbous enlargement called an *ampulla*. As the contiguous limbs of the superior and posterior vertical canals unite near their

### LECTURES TO NURSES ON ANATOMY.—*Continued.*

extremities to form a single canal, there are five and not six openings of the semicircular canals into the vestibule.

The semicircular canals, as we have already noticed, are placed behind the vestibule. In front of this cavity, and communicating with it, is another excavation called the *cochlea* (*k*), a mould of which, if such could be taken, would resemble the shell of a snail, the broad part being directed inwards towards the cranial cavity, and the tip or apex forwards and outwards in the direction of the front wall of the tympanum. The tubular chamber within this shell, which chamber is about one inch and a half in length, takes two turns and a half around a central axis of bone. This spiral chamber is divided into a double spiral tube by a septum or partition made up of bone, delicate cartilage, and thin membrane. Just at the tip of the shell the septum is wanting, so that the two passages communicate with one another.

We should endeavour to remember the names of these different parts enclosed within the snail's shell or *cochlea.* The central axis of bone around which the double chamber takes two and a half turns is called the *modiolus*; the septum or partition between the two divisions of the spiral chamber the *lamina spiralis*; the division which communicates with the vestibule the *scala vestibule*, and the other, shut off from the tympanum by a membrane stretched over a round hole called the *fenestra rotundum*, the *scala tympani.* Of these the most important part for us to take note of is the lamina spiralis, as on this partition are spread out the ultimate filaments of the nerve of hearing.

The inner wall of the vestibule is riddled like a sieve by very minute apertures through which pass those minute filaments of the auditory nerve (*l*) which are distributed to the contents of the vestibular cavity, and to the semicircular canals. The filaments of this nerve which are distributed to the cochlea, which filaments seem to be exclusively the nervous organs of hearing, enter the base of the cochlea, and before distribution over the lamina spiral are transmitted along a central canal which passes from the base to the apex of the modiolus.

The complicated system consisting of vestibule, cochlea, and semicircular canals is called the *osseous labyrinth.* This labyrinth forms a continuous, though very irregular, cavity, the bony walls of which are lined by a delicate membrane. Unlike the tympanum, from which it is completely separated by the thin membranes closing the fenestra ovalis and the fenestra rotunda, it is a water and not an air chamber, as it contains a limpid fluid called *perilymph.* Suspended in this fluid and anchored to the walls of the *osseous labyrinth* by filaments of the auditory nerve is an irregularly shaped and closed bag of thin membrane, which is known in contradistinction to the osseous labyrinth as the *membranous labyrinth.* This occupies the vestibule and the semicircular canals, and presents an exact counterpart of these chambers, the vestibular portion being represented by an oval bag called the *utricle*, communicating by five openings with three curved tubes which, though much smaller than the bony semicircular canals, are closely similar to these in shape, and present the same dilatations or ampullæ at their attached extremities, to which dilatations and not to the curved portions of the tubes are distributed the terminal nerve filaments. The membranous labyrinth is not only suspended in fluid—the *perilymph*—but contains and is distended by a similar fluid called *endolymph.* Attached to the utricle is a smaller closed bag called the *sacculus*, which like the larger bag is fixed by filaments of the auditory nerve and contains perilymph.

Although the membranous labyrinth and the structures enclosed within the cochlea are closely connected, being bathed by the same fluid, and supplied by divisions of the same nerve, each of these two parts of the internal ear, the so-called organ of hearing, has a distinct and quite separate function. Whilst it is generally acknowledged that the cochlea is concerned in the reception of auditory impressions, it has been asserted on very good authority that the membranous semicircular canals do not take part in the sense of hearing. These structures, it is now held, transmit through the eighth or auditory nerves those impulses called *labyrinthine impressions*, which enable the cerebellum to maintain the normal equilibrium of the body and a perfect adjustment of its muscles during their combined action in walking and standing.

### The Salary Question in the Colonies: Remarkable Government Document.

BY AN OCCASIONAL CORRESPONDENT.

THE unwisdom of nurses rushing to countries advertising large salaries, hardly needs to be demonstrated. But as one who has had a varied experience of nursing in the coast ports, China and India, the details I am in a position to furnish should not prove entirely valueless.

### High Salaries and Their Disadvantages.

Speaking broadly, high salaries represent unhealthy climates, high risks, and high prices. Under the first heading come such places as the West Coast of Africa and Rangoon. Perhaps it may be urged that the two first headings might have been incorporated, for obviously an unhealthy climate brings its own risks, but these may also be encountered in a so-called healthy station, where the soil, sanitation and general conditions may be entirely satisfactory, but where intercourse with other countries, commercial or otherwise, opens up numerous possibilities. Take for instance Shanghai in the present year; the newspapers have told how an epidemic of scarlet fever—that rare disease in the East—has ravaged all grades of society, and literally blotted out whole families. Look at Hong Kong, the junction of many trading routes, and in the same year note its epidemics of plague, cholera, small-pox and dengue fever which in many cases have been traced to the shipping. And similarly in all coast ports high risks may be encountered, although it is only fair to add that that immunity from disease enjoyed by nurses at home has a wider significance abroad.

### Payments for Food and Milk.

Upon the third heading there is much more to say. Some may contend that a high salary is worth many risks, and that one's expenditure is in one's own hands, but their horizons must have been limited, and their experiences only of an insular or continental type. They have never suffered the income-tax of India, or made acquaintance with the fluctuating dollar of the coast ports and Crown colonies. Having had her training in a home hospital, where everything (even to a beverage other than water) has been provided, a nurse would never dream of an allowance for food being embodied in her salary. Yet so it is, and at the end of a month or quarter the mess president, matron, or senior sister, presents each member of her community with a bill which varies according to season and locality. During the last six years I have paid as much as £50 per annum

### THE SALARY QUESTION IN THE COLONIES: REMARKABLE GOVERNMENT DOCUMENT—*Continued.*

and as little as £23. Drinks of all kinds are extra, and these constitute a considerable item when "aqua pura" is not available and recourse has to be made to "minerals," red wines, or milk. The latter is charged at 8d. or 10d. a quart, if cow's; less if buffalo's or goat's. Tropical veterans will urge whisky as a prophylactic measure against malaria, etc., but, somehow, the womanliness of woman seems to recede in the presence of spirituous liquor.

### Expenses of Coach Hire.

Another source of daily expenditure is the hiring of a jinrciksha, sedan chair, or ghari, for only in the early morning or late evening can walking be tolerated, and these hours are not always at a nurse's disposal. There are other minor ways of depleting the salary, but I will refrain from further mention for fear of appearing too pessimistic. It might easily be asked "Why then do people ever accept appointments abroad?" Well, for reasons as varied as those which prompt women to become nurses. And I must admit that there is a certain glamour in the life of the East which obliterates all its deficiencies. In what it lies it is difficult to define with exactitude, whether in its freedom from restraint, its perpetual sunshine, its camaraderie, its gay social life, or a mingling of all. At any rate, it is certain that when once nurses have entered upon work abroad they rarely return to home institutions, except to freshen their somewhat rusty knowledge of things surgical. The sense of restraint, the lack of sunshine, the lack of spontaneous bonhomie, and the much-broken-into off duty hours at home will prove irksome to the nurse who had almost forgotten that the visiting physician and his band of students ever existed.

### More Responsible Work.

Tropical hospitals are allotted barely sufficient doctors to do the work, so that unless urgent cases arrive between the hours of the morning and evening round, no visits are made by them. For this reason, too, sisters are not infrequently asked to administer chloroform—ether is rarely used in the East as it evaporates so rapidly under the influence of heat—a responsibility which they by no means appreciate. In fact, in many ways the position of sister is one of greater mental strain than at home, and were it not for the eight hours' uninterrupted daily leave she could scarcely withstand the exhausting heat or more trying humidity of the summer season. Yet in spite of all the foregoing, it is possible to enjoy existence and to save; two of the surest methods of doing so being the wearing of outdoor uniform and the purchase of a bill-file! The natives—Europeans, too, alas!—have an unhappy knack of sending in their accounts more than once, and are only content to forego further payment when confronted by a receipt. As I have hinted before, friends are easily made, and frequently when their leave is due they will ask as a favour permission to send over to a nurse a hack and groom or a pony and cart, esteeming it a privilege if one will personally keep them exercised. By this means vehicle hiring is dispensed with, and an enjoyable time at a low rate insured.

### Pensions at Government Hospitals.

Finally, and perhaps now I had better confine myself to Government hospitals, since the rules of others are so varied that they practically defy classification, there is a pension, which in India amounts to £50 per annum after 15 years' service. There too, a grateful country rewards the work of its faithful servants by a bonus of £60 (approximately) at the end of 10 years, or £30 at the end of five, if they decide against further re-engagement. This strikes one as being humane treatment, for it shows that the risks encountered—including the debility which manifests itself in tropical climates—have had recognition; a recognition evidenced in no other place, save the sick or life assurance offices. In unpleasing contrast with all this comes the action of the powers controlling the Crown Colonies, who have recently sent a circular note to all civil servants, proposing a salary scheme upon a sterling basis. The advantages of such currency would be speedily acknowledged if the amounts proposed were adequate to the present emoluments, but to be asked to reduce her own stipend by practically one-fifth, rouses the ire even of that self-contained individual, an English nurse. The suggested salary, according to the new scheme, is—minimum salary, £110; triennial increment, £20; maximum salary, £150; out of which, in addition to paying for food, the sisters will be required to find their own fuel and light, which, hitherto, in every hospital with which I have come in contact, has been furnished free. The nurses under agreements will be compelled to accept these terms at the end of their five years of service, so that a sister now being paid £162 per annum, out of which she only pays for her food, will be receiving considerably less than she enjoyed as a novice. Six months are allowed for contemplation of this proposal—six minutes would suffice for the average woman!—and at option sterling rates or the dollar rates under conditions not yet specified, will be paid. But—and here the iron enters into the soul—upon rejoining at the end of a term of five years the sterling rates will be compulsory. It sounds incomprehensible, especially as on account of greatly increased rates of living, higher salaries were recommended, were sanctioned at home, and have been received since 1900.

### Extracts from the Note.

Still, there is no getting away from official black and white, and I myself, during a recent stay in Hong Kong, read, marked, learned and inwardly digested this extraordinary document, of which the following extract may be interesting:—

". . . If the nurses elect to stay on a dollar salary, they must abide by such at present rates during the rest of their service in Hong Kong.

"The system of rate of exchange has not yet been fixed by the Secretary of State. At present it is the average from July 1st two years before to June 30th of the year before. It is suggested that the monthly rate be taken as in the case of exchange compensation.

"Officers at present serving under agreements have the option of coming under the sterling salary scheme, and will be required to accept sterling salaries on re-engagement."

Against the salaries to be paid to the nurses is marked "free quarters." Also, "these salaries include allowances for light and fuel and rations."

It is impossible to think that a reduction of salaries will be insisted upon; it would be against the British love of justice and would be putting a slight upon long service and valuable experience. One prefers rather to believe in a paternal Government who are simply proposing to act in this manner because "someone has blundered!"

### To Nurses.

We invite contributions from any of our readers, and shall be glad to pay for "Notes on News from the Nursing World," or for articles describing nursing experiences, or dealing with any nursing question from an original point of view. The minimum payment for contributions is 5s., but we welcome interesting contributions of a column, or a page, in length. It may be added that notices of appointments, entertainments, presentations, and deaths are not paid for, but that we are always glad to receive them. All rejected manuscripts are returned in due course, and all payments for manuscripts used are made as early as possible after the beginning of each quarter.

### Words of Advice to Nurses.

BY ESTHER H. YOUNG.

### LECTURE III.—POINTS OF GENERAL CAREFULNESS.

Now let us consider some points of general carefulness necessary to our well-being as women, and some of them particularly necessary for nurses to remember.

No one, unless really careful, can make a good nurse. Nursing is caring for other people—not just making their beds, taking their temperatures, and applying remedies—it is caring for them, body, soul and mind.

To train ourselves to care for others we must take reasonable care of our own selves, body, soul and mind. And, too, just as we must take care of the property or belongings of our patient, so we must take proper care of our own, and also (I am afraid nurses too often forget this!) we must be very careful in the use of everything provided by the hospital.

First, the Nurse's own health.

Though a nurse, by reason of her calling should be ready to suffer, and to endure discomfort or pain, and even if necessary, to lay down her life for her patient, yet it is her plain duty to take reasonable care of her own health, for the sake of her patients, and as part of her duty towards God, who gives all life and health, and expects that life to be taken care of. As suicide is distinctly sin—the sin of murder (our lives are not our own to take)—so it is sin in a lesser degree so to tamper with health as possibly to shorten or injure life. Nurses are very apt to be careless about their own health in various ways. They are almost proud of this fact, and boast of not "bothering about themselves." They forget that no work is properly done without due rest and care, or with the body in an unfit condition, and even raw little "pros." fancy there is a special merit in "hanging on." But the more advanced we get in our work, especially in nursing, the more we need to remember this fact: That we are not at all indispensable, somebody else can so easily be found to do our work. When we struggle against going off duty and leaving our little bit of work to others quite as competent as ourselves, instead of showing (as we think!) much courage and a strong sense of duty, we really show a lack of prudence and much conceit. Those who are in training in hospital should try to remember that those in authority over you are bound to care for you, are responsible for your well-being, and that you materially increase their anxiety and by no means lessen their responsibility (as you sometimes appear to think) by hiding from them the fact of any indisposition, however slight the nature of it, either really or only in your own estimation.

Don't "kick against" the sick-room, in your imagination terrible, though in reality a very excellent resting-place for you. Indeed, to go there is an almost necessary part of your training.

Speak of little ailments at once. Don't hang on because you don't want to leave a certain ward or a particular case, or because there is a whole or half-day "due" in the way. Don't say, when asked whether there is not something wrong, that you are "quite well," when you know there is a pain or swelling somewhere. This is not bravery, but lack of common sense and even of strict truthfulness. You are practically asked, "Is God's hand upon you?" You say "No." Is not that the way to get it unmistakably laid upon you?

Ah! those fingers! I suppose nurses must get them, even when they are careful; but by speaking about them at once much trouble may be avoided. You think your matron or home sister a real dragon for seeing those "done up fingers" at breakfast time, and insisting on "Off duty, nurse!" don't you? But if you had seen as many poisoned fingers and the bad results that follow when neglected you would not wonder at the vigilance.

Speak at once and avoid "lumps under arms," loss of a limb, or having to give up work altogether. No! I am not exaggerating, but recalling facts.

When you have cut or pricked your finger, and forgotten to cleanse and cover it at once, and a white spot appears, don't ask advice from various people and get "haphazard" fomentations, but show it to the right person at once.

"Throats." Don't take a little of 3's medicine and use 5's gargle, report yourself at once.

"Swollen feet." Don't put them in "easy" shoes and "keep on" on at your work, show them at once.

Joint pains, headaches, coughs, indigestion—you think you can manage them all, don't you? Perhaps, but with the result probably, that in the end you are off duty for weeks or months, instead of days. Don't take any drugs at all, unless ordered by a doctor. Promiscuous medicine taking is a very bad habit, and one which nurses, unless they are careful, are very apt to get into. It usually starts in hospital days. From time to time we hear of a nurse losing her life from taking an overdose of medicine, accidentally (or, sad to say, intentionally), and it is so often found to be the result of a bad habit started in her "pro." days.

The temptation no doubt is great, and, like all other temptations, once yielded to, it soon gets beyond your power of self-control. I cannot too strongly put before you the great danger and grave consequences of the practice of dosing yourselves or one another. It is just as wrong as it is to yield to the temptation to take alcohol in excess.

Granted you are quite well, take no drugs, and are possibly a "total abstainer," still your body needs care, special care, because of your calling as a nurse and of the life you lead in hospital. Therefore let me, after some years of experience, offer to you who are perhaps just entering upon hospital life, some practical suggestions.

Clothe yourself wisely. You are often in very different temperatures as you leave a warm ward to go through passages or out of doors to another ward or your room, therefore wear flannel next your skin always, summer and winter. Take care of your hands. Poor little "pros." think it is quite part of being a nurse, that because they have rough work to do their hands must become rough and hard, and so unfit to touch sick folk. No, little one, not if you take care to keep your nails quite short and most scrupulously clean. Sometimes poisoned fingers start from under the nail. In well-groomed nails this would be impossible; scrub them constantly with a hard nail-brush; don't let any dirt remain under the nail; use a pin or hair-pin to get it out.

Never under any circumstances bite your nails. Besides being an ugly habit, you may by so doing contract disease. Always when nursing any infectious case, wash and scrub your hands and nails before eating any food (even sweets!). The neglect of this habit is undoubtedly often the cause of nurses contracting infectious disease.

Wash out your mouth with some disinfectant when nursing infectious diseases, especially diphtheria.

Take care of your hair. Your caps are given you to protect your heads, therefore have as little hair as possible outside those caps. Fringes, or anything approaching them, are an abomination, so is floppy hair, in front or behind, in nurses' uniform. When an untidy head is noticed, the excuse invariably is "I washed it last night." Yes, no doubt, but so do others wash theirs, and yet they are able to appear minus fluff!

*(To be continued.)*

### Everybody's Opinion.

[Correspondence on all subjects is invited, but we cannot in any way be responsible for the opinions expressed by our correspondents. No communication can be entertained if the name and address of the correspondent are not given as a guarantee of good faith, but not necessarily for publication. All correspondents should write on one side of the paper only.]

### FORRES LEANCHOIL COTTAGE HOSPITAL.

"The Secretary of the Forres Leanchoil Hospital (Mr. Robert Urquhart, jun.)" writes: I observe in your publication of 1st inst. a notice as to this hospital, in which you mention that the managers offered Miss Reid, the retiring matron, a sum of £20 to withhold from publication an apology by one of the medical gentlemen. I shall feel obliged if you will state in your next publication that there is no foundation whatever for this statement. It first appeared in the *Aberdeen Journal* of 14th ult., and it was contradicted by me in that newspaper, and it is unfortunate for our hospital that this contradiction did not come under your notice.

[As Mr. Urquhart surmises, the contradiction did not come under our notice, but we are very glad to hear that the statement made, not by us, but, according to the *Aberdeen Journal*, by the late matron, was unfounded. We regret, however, that the secretary is not, apparently, in a position to give any assurance that affairs at Forres Leanchoil Cottage Hospital have been put on a satisfactory basis, although we observe that a new matron has been appointed.—Editor, Hospital.]

### VILLAGE NURSES IN CUMBERLAND.

"Miss Amy Hughes" writes from St. Andrew's House Mortimer Street: While quite agreeing that fully-trained nurses are the best for the sick poor when available, may I point out a fatal flaw in the well-intentioned plea of "A. B.," in her letter of November 1st, in favour of only employing them, viz.—that the hospital-trained nurse is able to decide from her diagnosis of a case whether medical assistance is needed or not. It is not the province of any nurse, however highly trained and experienced, to diagnose cases. That is the work of the qualified medical man, and his alone. From "diagnosing" to "prescribing and treatment" is an easy step, and an open door to the worst forms of quackery. There is a sharply-defined line between the province of the nurse and the work of the doctor, and nurses as a body must learn to respect it, or lower their standard of honourable service in a vain endeavour to become unqualified assistants. This idea of "diagnosis" is a real danger in district work, and must not be entertained for a moment. I may add that the village nurses, being certificated midwives, can take normal maternity cases alone, if desired. I have no wish to enter into the questions raised with regard to the Cumberland Nursing Association, but would like to enter a protest against the special argument used in favour of trained nurses.

### WORDS OF ADVICE TO NURSES.

"Sister Elizabeth" writes: Concerning the statement made in "Words of Advice to Nurses" that "the probationer of to-day is wanting in wholesome awe of her seniors," it seems rather unfair to lecture the nurses and probationers so publicly for failings which it is quite within the power of any matron or sister to correct during the long period of training to which probationers are submitted nowadays. As a sister with five probationers under me I can only say that I feel very sorry for any matron or sister who owns to such a difficulty. In some hospitals it is no uncommon thing for a sister to correct or scold a nurse before the patients. Now, to a high-spirited or a sensitive girl this is a needless humiliation. From a high-spirited girl you will get an answer back. In the case of a sensitive girl you will encounter scathing looks from the patients, who are sure to take her part. In either event the sister must feel herself in an undignified position, while, had the reproof been given in private—well, from my experience it would not have been needed a second time. On entering a fresh ward for the first time, let the sister tell the probationer her customs with regard to sitting, standing, speaking, etc. I am sincerely glad to see that the question of stimulants is mentioned. When a nurse resorts to these it is a sure sign that she is needing medical treatment. Probably a tonic and a day or two's rest, if taken in time, would save years of misery to herself and friends. If a nurse is looking ill, does not sleep, or has neuralgia, let her see the doctor. There will not then be any difficulty about stimulants. It must be remembered, however, that according to hospital etiquette, a nurse may only see a doctor for treatment with the matron's consent.

### THE ROYAL RED CROSS AND ITS RECIPIENTS.

"An Onlooker" writes: Most people have the mistaken idea that to gain the Royal Red Cross one must necessarily have performed some deed of daring, or faced some kind of danger in the performance of one's duty, and I believe that originally it was intended for this purpose. In reading over the names of the recipients during the present campaign I have looked in vain for some mention of women I have heard of as having gone through great privations and dangers when the camp they have been in happened to be attacked by the Boers or shelled; but no notice, it seems, is taken of such people. Then, there are numbers who have given their lives for their country, and this also is counted but a small thing when compared with the work done by a number of idle women seeking fresh excitement. These people came forward with offers of assistance, and some were even allowed to help in the hospitals at the front. In one or two cases they did real nursing, but these were not the decorated ladies. Generally speaking, they arrived in the wards or tents about 11 a.m., bringing small comforts for the men, which had been sent out either by the Red Cross Society or private firms; if there were any doubt about a patient in one of the tents having anything they thought infectious, that tent would be treated by these brave ladies as though it were plague ridden. Others who have been recommended by their commanding officer as having in his judgment worked so well as to have deserved special notice, in many instances have been passed over as undeserving cases. How then are the cases judged, and who has the final decision? If the Commander-in-chief, he should surely be the first to recognise the claims of nursing before those of favouritism. That all who hold superintendents' posts should receive it as they did, seems a doubtful claim to special merit when one considers that those doing the harder and more dangerous work were left out. Then at home again and again it has been given for outside help in providing convalescent homes for officers and colonials. One does not grudge the recognition of these services in some form or other; but that a distinction which was first intended for nurses, and nurses only, should be given seems like awarding the V.C. to a man for saving life either from fire or water, and one never hears of this being done.

### THE CHANGES IN THE ARMY NURSING SERVICE.

"Another Reserve Sister" writes: The impending changes in the nursing of the army seem likely to be of little benefit either to the service generally or military hospitals in particular. The regulations as at present issued give us only a skeleton plan, with few or no instructions as to how this plan is to be made into a living and working body. Under the new scheme the matrons' duties (on paper) seem to be very like those performed by superintendents at present, but in her hands, I take it, will rest the task of piecing together, starting, and keeping in order the new machine. But it is the sisters' position which will be most affected. In the multiplication of her duties the sister will have little time to spare for actual nursing. It will take her all her time to keep her equipment together, to write diet summaries and linen inventories, to see to the cleaning, warming, lighting, and ventilating of the wards and annexes, and last, but not least, to keep the nurses and orderlies working together without friction; not to mention the drawing out and handing in of the personal and hospital kits of the patients. In fact she will be a wardmaster and little else. This last rule with regard to the kits will be found the most unworkable of all. It takes a soldier to deal with soldiers, and it seems hardly fair to ask women of refinement to perform such duties as this. Responsibility is thrust upon the sisters, and no rank or authority given them wherewith to uphold it; in fact their position will be infinitely lower in the eyes of the orderlies and patients than that which they formerly occupied. Granted, the army is mainly composed of men, who, as a high authority put it, are heroes when fighting and gentlemen on other occasions, yet there are black sheep in every flock, and what protection will the sister have from being taken advantage of by lambs of this complexion? Even the pack storekeeper, though generally a senior noncommissioned officer, finds difficulty in dealing with some of them. Surely the making inventories of soldiers' uniforms and soiled linen, and the dragging about of kit bags, is no work for women, nor by any stretch of imagination can it be called nursing the sick. It is impossible to see who will benefit in the very least by this arrangement. Why saddle the unfortunate sisters with work which someone else could do equally well, and probably far better? The position of the staff nurses—when they are found—will be a very ambiguous one indeed. They will apparently be on the same level as the orderlies, and share the ward work between them. Let us trust that all heroic and gentlemanly qualities will be well to the front in this partnership of labour. I must not ask for further space in your columns, and my apology for writing at all is the disappointment I feel in common with many of my friends at the sweeping changes to be made in a service in which we had hoped to apply for posts.

### Appointments.

[No charge is made for announcements under this head, and we are always glad to receive, and publish, appointments. But it is essential that in all cases the school of training should be given.]

Ashton-under-Lyne District Infirmary.—Miss Florence Amelia Haig-Brown has been appointed matron. She was trained at St. Thomas's Hospital, London, and has been since night superintendent at Brompton Hospital. She has also been sister at St. Marylebone Infirmary, and since August, 1896, sister at the Nightingale Home, St. Thomas's Hospital.

Blackburn Infirmary.—Miss Mary Brunton has been appointed sister. She was trained at Brownlow Hill Infirmary, Liverpool, and has since been sister at the Ear and Throat Hospital, Birmingham.

Cannock Workhouse Infirmary.—Miss C. M. Heafield has been appointed head nurse. She was trained at the Withington Hospital, West Didsbury, Manchester, and has been charge nurse at Aston Workhouse Infirmary for two years. She holds the L.O.S. certificate.

Cumberland Infirmary, Carlisle.—Miss Maud Burgess has been appointed sister. She was trained at Oldham Infirmary, where she has since been staff nurse and temporary sister. She has also been private nurse for the Scottish Nursing Association, Edinburgh.

Forres Leanchoil Hospital.—Miss Margaret Henderson Levack has been appointed matron. She was trained at the Northern Infirmary, Inverness, and has since been nurse at Govan Parochial Hospital, Glasgow.

Frere Hospital, East London, South Africa.—Miss Jessie Thorne has been appointed matron. She was trained at the London Hospital, England, and has since been private nurse in East London for three years. She holds the L.O.S. certificate.

Great Yarmouth Workhouse Infirmary.—Miss Grace Wood has been appointed charge nurse. She was trained at Camberwell Infirmary, where she has also been staff nurse. She has since been staff nurse at the Gordon Hospital, Vauxhall Bridge Road, London.

Hundon District Nursing Association.—Miss S. Nicholls has been appointed district nurse for the village of Hundon, in Suffolk. She was trained at the Royal Southern Hospital, Liverpool, and was employed at the Deaconness Nurses' Institution, Chester, for 22 years, where she did both private and district nursing.

Leeds Union Infirmary.—Miss Elizabeth A. Gittins has been appointed matron. She was trained at the Birmingham Union Infirmary, where she has since been home sister and assistant matron.

Mercers' Hospital, Dublin.—Miss Jane H. Forde has been appointed night sister. She was trained at the Royal City of Dublin Hospital.

National Hospital for Diseases of the Heart, Soho Square, W.C.—Miss E. A. Barber has been appointed matron. She was trained at the Western Infirmary, Glasgow, and the Glasgow Maternity Hospital. For the last 18 months she has been sister at the National Hospital for Diseases of the Heart. She holds the L.O.S. certificate.

New Infirmary, Shirley Warren, Southampton.—Miss Kate Oyler has been appointed night superintendent. She was trained at Guy's Hospital, and was afterwards sister at the National Hospital, Queen's Square, London. She has also been sister of the theatre and out-patient department at the Royal Portsmouth Hospital, and sister of the women's surgical wards. Subsequently she was matron *pro tem.* at the Surbiton Cottage Hospital, and has since been sister at the Stanley Hospital, Liverpool. She holds the L.O.S. certificate.

South African Constabulary.—Miss Elizabeth Fry and Miss Catherine Terry have been appointed nursing sisters on the South African Constabulary, and posted to A Division, Potchefstroom, Transvaal. Miss Fry was trained at the Meath Hospital and County Infirmary, Dublin, afterwards serving on the private nursing staff of that institution. She was then night sister for 18 months at Sheffield Royal Infirmary; joined the A.N.S.R. 1900, and worked at Aldershot, Kimberley and Charlestown until peace was declared, when she joined the S.A.C. Miss Terry was trained at the Evelina Hospital for Sick Children and King's College Hospital, London. She was afterwards ward sister at Moorfields Eye Hospital; joined the A.N.S.R. March 1900, and worked at Orange River, Kimberley and Charlestown until peace was declared, when she joined the S.A.C.

### Presentations.

Albert Infirmary, Winsford.—Last week Miss James, who is resigning the position of matron of Albert Infirmary, Winsford, owing to her approaching marriage, was presented with a silver tea-service by the Board of Management. Miss James also received gifts from the nurses and the servants.

### Death in Our Ranks.

We regret to announce the death of Sister Florence E. Jones from enteric fever, which took place at the Greek Hospital, Alexandria, on November 1st.

### Presentation to Miss Annie Hobbs.

On Tuesday afternoon a pleasant social meeting of members of the Nurses' Co-operation took place at the Howard de Walden Club, where a presentation to Miss Annie Hobbs, who has been appointed Secretary to the Nurses' Auxiliary Society, was made. The nurses gathered in the Residents' Room about four o'clock, and a little later, Miss Roberts, Lady Superintendent of the Co-operation, having arrived, the little ceremony was performed by Miss Ward and Mrs. Brown, each of whom said a few words about the pleasure the occasion gave the nurses, the regret they felt at losing Miss Hobbs, and how they all wished her every happiness in the new work she had entered upon.

Miss Hobbs, with her pretty present—a tea-service—on the table before her, said that what she felt most inclined to do at the moment was to make tea at once and entertain all the nurses.

"I shall never find words," she continued, "in which adequately to express my appreciation of this magnificent gift. I do thank you, not only for the gift, but so much more for the kindly feeling of which I know it to be the expression." Miss Hobbs went on to observe that she could never forget the happy time she had spent among the members of the Co-operation during the last three years as the lady superintendent's assistant; they had treated her with the utmost courtesy and consideration, and had made her feel that they looked upon her as their friend; it was not without many regrets that she left them. Still, she would not be far away; and although no longer belonging to them, she hoped that they would still let her be their old friend, and come and see her when they could; she would be just as pleased to welcome them at 10 Orchard Street, as she had always been at 8 New Cavendish Street. Miss Hobbs concluded by asking the nurses present to tell any who were prevented from being there, and whom they might meet, how delighted she was, and how she reciprocated the kind wishes for future happiness that had been expressed. She hoped that all the members would come and see her at the Auxiliary Nurses' Society's office, so that she might thank them personally.

The nurses, having applauded Miss Hobbs for her little speech, crowded round to examine the dainty Coalport Leadless Glaze teacups and saucers and plates, and the silver tea or coffee pot, with an inscription on the bottom: "Presented to Miss Annie Hobbs by the members of the Nurses' Cooperation, as a token of esteem and affection, November 11th, 1902." The teapot, with sugar and cream jug, rested on a small wood tray, and there was, in addition, a silver kettle on a spirit stove. In a dainty book, with an artistic design on the cover, were the names of those who had subscribed to buy the gift. This pleasant little function over, the nurses adjourned for tea, and broke up into groups.

Miss Hobbs is already at work. She is fond of office-work, and devoted to nurses and their interests, and she looks forward with pleasure to doing a useful service for those whose age precludes them from membership of either the Nurses' Co-operation or the Chartered Nurses' Society. The Auxiliary is now on the telephone, and the new secretary hopes to get many calls through it for the nurses on her list.

### Wants and Workers.

Nurse Williams, Broadway, Worcestershire, thanks all who so kindly sent her offers of The Hospital, one of which she has accepted.

### For Reading to the Sick.

"LORD, STRENGTHEN US."

Lord, strengthen us; lest fainting by the way
We come not to Thee, we who come from far;
Lord, bring us to that morrow
Which makes an end of sorrow,
Where all saints are
On holyday.

Where all the saints rest who have heard Thy call,
Have risen and striven and now rejoice in rest:
Call us to home from sorrow
To rest in Thee to-morrow;
In Thee our Best,
In Thee our All.

*C. Rossetti.*

If Christ showed His faithfulness by dying for us, we shall count it all joy to have our faithfulness tested by following Him in the reproach, the weakness, the loneliness, of the way of sorrows. There is a deeper joy in faithfulness to the Crucified than can possibly be our portion in the way of the world's honours. . . .

How prone we are to shrink from the cross, and yet the cross is the school of Divine teaching. None can learn anything of God truly in any other way. All other knowledge is but head-knowledge. Knowledge gained in the discipline of the cross is heart-knowledge, full of blessed experiences of heavenly inspiration.

We are weak until we begin to suffer. As we continue in suffering with Jesus, we find the strength of faithfulness beginning gradually to develop. It is a law under which we are born that suffering strengthens character and brings the various faculties of our nature into play. As we triumph over continued difficulties, we gain the grace of perseverance.—*Benson.*

It may be often a very obscure life, and the events that make it up such as do not meet the eye or the ear of any but the narrowed circle. Its duties may be homely, its victories the most commonplace—that is, in the judgment of men; not so in the sight of God. He is no respecter of persons, and the hidden story of the lowliest among His children ranks with the most notable of public histories. Nothing is lost, "We shall find it all," says a loving soul, "a thousand times treasured up for us—each earthly hope which we surrender, because we have a better hope above, when we by His grace have reached the shores of that distant land, where trial and sorrow are over, when Heaven itself shall open before our enraptured gaze."—*Canon Tetley.*

"Heavenwards, Homewards! through the dense
Dark clouds of sorrow, and the sense
Of present frailty, past offence;

Heavenwards, Homewards! by the road
The poor in spirit ever trod,
And tread, in pilgrimage to God

Heavenwards, Homewards! till they win
That blest inheritance, wherein
Is no more sorrow, no more sin."

*S. J. Stone.*

### Echoes from the Outside World.

### The King's Birthday.

THE King celebrated his sixty-first birthday on Sunday, and received from all parts of the world telegrams and letters of congratulation. Among the numerous demonstrations in connection with the event was the lighting on Saturday night of a great bonfire, some 40 feet high, on the eastern slope of Alexandra Park. The flames could be seen for miles around. His Majesty's list of birthday honours included the creation of five new Privy Councillors, eight new baronets, and twenty-one new knights of whom Sir R. H. Borwick is well known in connection with philanthropic work, and Sir Robert Hensley is chairman of the Metropolitan Asylums Board. There are three new Knights Commanders of the Bath, many additions to the Royal Victorian Order, and the appointments to the Imperial Service Order which the King recently instituted for members of the Civil Service of the Empire as a recognition of long and meritorious service are very numerous. The only lady thus singled out for distinction is Miss M. C. Smith, superintendent of the Post Office Savings Bank Department.

### The German Emperor's Visit.

THE day of the German Emperor's arrival in England was cloudy in the early morning, and later on the weather degenerated, so that when his Majesty inspected the 1st Royal Dragoons—of which he is Colonel-in-Chief—at Shorncliffe, it was in a driving storm of sleet and rain. Notwithstanding this the Kaiser made a stirring little speech to the men in which he complimented them on their "splendid appearance on parade," and on the "faultless manner" of their ride past and called for three cheers for King Edward VII. The Emperor then lunched with the officers in the mess, afterwards leaving by special train for Sandringham. He proceeded by way of Liverpool Street Station where a fresh engine was attached to the train, but every avenue of approach was guarded by police, and no one but the officials saw his Majesty. At Wolferton Station he was met by King Edward, and the two Sovereigns shook hands most cordially and embraced. On Sunday the King and Queen, their Royal guest, and most of the house party, which included the Prince of Wales, the Bishop of Ripon, Earl and Countess Roberts, Mr. and Mrs. Chamberlain, and Mr. Balfour, attended Divine Service. In the evening there was a dinner-party and Herr Gottlieb's band was in attendance, and Herr Kubelik was present by command. On Monday morning there was wild duck shooting on Wolferton Marsh, the Emperor and the Prince of Wales going by motor-car, and in the afternoon trees were planted by the King, the Kaiser, the Prince of Wales, Prince and Princess Charles of Denmark, and Princes Edward, Albert, Henry, and Princess Victoria Mary of Wales respectively. The avenue in which the trees were planted will for the future be called "Kings and Queens Avenue." Tuesday was mainly occupied with partridge shooting at Dersingham.

### Royalty and Needlework.

AT the end of last week the garments and other articles made and collected by the members of the London Needlework Guild during the past year were exhibited in the north gallery of the Imperial Institute. The total number sent in exceeded 50,000, and on the whole they were of better quality than in 1901, although not quite so numerous. The Princess of Wales—who succeeded the late Duchess of Teck, her mother, as patroness—is again the largest contributor, as many as 12,300 articles being forwarded by Her Royal Highness. This wonderful total includes 59 articles of men's clothing given by the King, some well-stuffed pillows made by the Prince of Wales, six woollen petticoats made by the Princess, a number of woollen comforters worked by Prince Edward and Prince Albert of Wales, besides other articles purchased by them out of their pocket money, and two pairs of cuffs knitted by Princess Victoria of Wales, who has thus early in life started on good works. The garments were this week apportioned and distributed. Nearly all the London hospitals, as well as some of the missions, prisons, refuges, and poor parishes of London participate in the gifts.

### Ministers at the Guildhall.

ONE of the most interesting features in connection with the Lord Mayor's Show on Monday was that the Prime Minister and the Colonial Secretary, returning from Sandringham, got blocked in Queen Victoria Street. Alighting from their carriages Mr. Balfour and Mr. Chamberlain accepted offers of stools, and, standing on them, watched the procession pass. On their return to their carriages they were loudly cheered. The Prime Minister was the principal guest at the Lord Mayor's dinner which followed the Show. The new Lord Mayor, Sir Marcus Samuel, made a very interesting announcement at the Guildhall, namely, that the King had graciously consented to open in person the new out-patients' wing of the London Hospital, and would be accompanied by the Queen. In the course of the brilliant speech in which Mr. Balfour responded to the toast of "His Majesty's Ministers," he referred to the King's illness, the Coronation, and the re-establishment of peace as the most important events of the past twelve months. He dwelt hopefully on the prospect in South Africa and with respect to foreign affairs, he strongly deprecated "the wild and fantastic inventions, of which, in certain quarters, the visit of the German Emperor had been made the text." In a striking passage, he said that he did not think the statesmanship of the world would be found unequal to the great task of maintaining international peace.

### Women and Drink.

AT a Temperance Meeting held at the Church House, Westminster, amongst the speakers was Sir Thomas Barlow, Physician to the King. He said that notwithstanding much was done to promote temperance, the broad fact remained that intemperance was one of our greatest national crimes, and the greatest hindrance to our national efficiency. Sometimes amongst women it was due to lack of occupation, but more often to trouble or worry. Or intemperance was often begun by those suffering from weakness and wearing pain because they found that alcohol gave temporary relief. Drunkenness, however, though it became a disease, started with self-indulgence, and should be treated as a sin. Dr. Barlow asserted that no serious alcoholic disease could be recovered from under twelve months. Rest and removal of the cause were the best cure, not drugs. He besought his hearers, in such cases, to get doctors and nurses who were teetotalers, and to form about the person, as it were, a sort of guild of protection, by sympathy, encouragement and example. It was all rubbish to say that female drunkards were never reclaimed. Inebriate homes and the Salvation Army knew better. The Bishop of London, later on, alluded to the rich whom he had seen ruined by alcohol and morphia, and told the following story: "A lady who had fallen into the morphia habit put herself under the care of a nurse, and craving for morphia sent an order to Ireland for a large parcel of dress goods. Before letting her have the parcel the nurse tested it with the Roentgen rays and discovered a bottle of morphia in the centre."

### Notes and Queries.

**The Editor is always willing to answer in this column, without any fee, all reasonable questions, as soon as possible.**

**But the following rules must be carefully observed:—**

**1. Every communication must be accompanied by the name and address of the writer.**

**2. The question must always bear upon nursing, directly or indirectly.**

**If an answer is required by letter a fee of half-a-crown must be enclosed with the note containing the inquiry, and we cannot undertake to forward letters addressed to correspondents making inquiries. It is therefore requested that our readers will not enclose either a stamp or a stamped envelope.**

*L.O.S. Certificate.*

(47) I have lost my L.O.S. Certificate, which I obtained in January, 1899. Can you tell me how I can get it replaced?—*M. L. P.*

Write and state your case to the Secretary of the London Obstetrical Society, 20 Hanover Square, W.

*Abroad.*

(48) Will you kindly tell me the best way in which I can hear of a patient wishing to go to South Africa in charge of a trained nurse? What nursing association on the co-operative system is mostly in want of trained nurses?—*Nurse O.*

You can only hear of a patient through advertisement. The Nurses' Co-operation, 8 New Cavendish Street, Portland Place, W., is the largest of its kind.

Will you kindly let me know to whom to write for information respecting appointments as assistant matron or home sister in lunatic asylums either in South Africa or New Zealand?—*Vagus.*

You might apply to the Inspector of Asylums, W. J. Dodds, Valkenberg Hospital for the Insane, Mowbray, Capetown, for information respecting South Africa. You will find in "Burdett's Hospitals and Charities" a list of the chief asylums in New Zealand; select the one you prefer and write to the Medical Superintendent.

*Hospital Training.*

(49) I am very anxious to enter a hospital for a regular course of three-years' training as I find what a bar to success the want of such a certificate is. I am 30 years of age, and have had eighteen months nursing in a large workhouse infirmary, eight months as head nurse in a home for incurables, and I also hold the L.O.S. certificate. I am an excellent traveller and should not mind where I went if I could get work.—*Eilitto.*

Yours appears to be a singularly unfortunate case. With the training and experience which you already have you should be able to earn your living easily, even though the more responsible nursing posts are closed to you until you gain the three-years' certificate. An advertisement might bring you in touch with a matron who would be able to utilise your services for the three years, and at the same time give you the opportunity of studying for and attending the examinations of the training school she superintends.

Can you give the names of any hospitals where the nurses are only on duty eight hours a day? I am willing to pay for the training received.—*Colonial.*

You will find full particulars in the "Nursing Profession: How and Where to Train." You might write to the Matron of the London Hospital, Whitechapel, E.

*Books.*

(50) Will you kindly tell me if a "Practical Text-book on Midwifery," by Robert Jardine, and a "Manual of Midwifery for the Use of Students and Practitioners," by Fothergill, are used for the examination? If not, what book shall I require?—*V. M. L.*

You do not say what examination. If it be the L.O.S. your teacher will most probably select the text-books.

I should be glad if you can recommend a good book on private nursing, giving hints to one commencing private work.—*E. M. F.*

It is doubtful whether a good book of the kind is published. After capability, adaptability is the most essential requirement. It therefore would be impossible to lay down any hard-and-fast rules.

*Massage.*

(51) Can you kindly tell me where I could learn Swedish massage in England?—*Nurse H.*

Write to the Secretary, the British College of Physical Education, Lancaster Gate, W.

Can you kindly inform me where I could get a thorough training in massage in Manchester, with certificate when proficient?—*Inquirer.*

There is no public course of instruction for massage in Manchester; there are private teachers with good references, but we cannot give their names. Write to the Secretary of the Society of Trained Masseuses, 12 Buckingham Street, Strand, W.C.

I should be glad to know particulars of the examination which has to be passed in order to be admitted into the Incorporated Society of Masseuses?—*H.*

Write to the Secretary of the Society, 12 Buckingham Street, Strand, W.C.

*Afloat*

(52) I am a fully trained nurse, and wish to get a post on board a ship going to South Africa or Australia. Would I have to go as stewardess, and what salary could I expect?—*Nolens.*

You had better write to one of the great steamship companies in London or Liverpool.

*Private Case.*

(53) I am a fully trained nurse with medical, surgical, mental, and massage qualifications, and I desire to receive into my home a delicate child with spine or hip disease, but I do not know how to get such a case, or what reference to give. I could take an imbecile child, and thought that £1 a week was not too much to ask. —*Nurse May.*

You must advertise for what you want, and you must give references from medical men as to your training, and from employers as to your competence, character, and kindness. If all are satisfactory, £1 a week is a very moderate fee to ask for the trying cases you seek.

*Cottage Hospital.*

(54) Can you tell me the name of some cottage hospital in Northumberland, or near Newcastle-on-Tyne?—*F. W.*

There is the Thomas Knight Memorial Hospital at Blyth. Lucy Eden Cottage Hospital, Bishop Auckland; Mary Hewitson Cottage Hospital, Keswick, and Jubilee Cottage Hospital, Penrith, are not far away.

*Maternity.*

(55) Will you kindly give me the names of institutions where maternity training is given at which there is no age-limit?—*R. O.*

You had better select a private institution advertising in our columns. All the hospitals have an age-limit.

Will you kindly let me know through your columns the best training school where I could train for midwifery without having to pay fees?—*E. S.*

None of the training schools take probationers without fees. Sometimes a private home advertises for a non-paying pupil, but she is very justly expected to give an equivalent in service.

How soon would it be quite safe for a lying-in patient to take a warm bath? and also how do you prepare sweet whey?—*Diragfarn.*

1. The medical man or midwife will tell you when to give the bath. 2. Whey is made by curdling milk with either buttermilk, or lemon-juice, or wine or rennet. The milk is warmed and the curdling agent put into it, and it is left in a warm place until the curd is set; then the whey is strained off, sweetened and flavoured according to taste. See any good cookery-book for the different varieties.

Will you kindly tell me if there is any method of obtaining a midwifery certificate by examination without going into an institute to reside for training?—*L. B. F.*

Write to the Secretary of the London Obstetrical Society, 20 Hanover Square, W., for syllabus of examination. You will be obliged to have practical as well as theoretical training, but arrangements might possibly be made for that privately with a local medical man.

I am a certificated monthly nurse, and should be very glad if you would tell me the best way of getting work.—*W. P.* and *N. W. P.*

You can only obtain a connection by obtaining introductions to medical men and patients through friends, or by advertisement.

**Useful Handbooks for Nurses.**

"Nurses' Dictionary of Medical Terms." Cloth, 2s.; leather, 2s. 6d.; post free 2s. 8d.

"On Preparation for Operation in Private Houses." 6d.

"Hospital Sisters and their Duties." 2s. 6d.

"Medical Gymnastics, including the Schott (Nauheim) Movements." 2s. 6d.

"The Human Body." 5s.

"Practical Handbook of Midwifery." 6s.

"A Handbook for Nurses." (Illustrated.) 5s.

"Tendencies to Consumption: How to Counteract Them." 2s. 6d.

"Syllabus of Lectures to Nurses." 1s.

The above works are published by the Scientific Press, Ltd., and may be obtained through any bookseller or direct from the publisher, 28 and 29 Southampton Street, Strand, London, W.C.

## Figures and Tables

**Fig. 64. f1:**
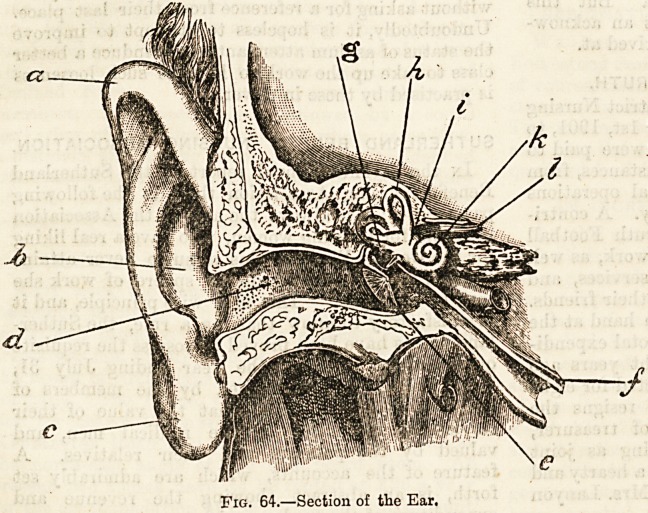


**Fig. 65. f2:**